# The relationships of parenting styles, self-esteem and rejection sensitivity: a latent profile analysis

**DOI:** 10.1186/s40359-026-05240-8

**Published:** 2026-08-01

**Authors:** Ling Guo, Zilan Ye, Yonglin Huo, Xinyi Wu

**Affiliations:** 1 Faculty of Psychology, Beijing Normal University, Beijing, China; 2 Henan Experimental High School, Zhengzhou, China; 3 Department of Psychology, Guangdong Medical University, Dongguan, China; 4 Shenzhen Futian Academy of Educational Sciences, Shenzhen, China; 5 The Third People’s Hospital of Foshan, Foshan, China

**Keywords:** Parenting style, Self-esteem, Rejection sensitivity, Latent Profile Analysis

## Abstract

**Background:**

A large number of theoretical reasoning and empirical studies have shown that both parenting style and self-esteem have an impact on the formation of rejection sensitivity, and there may be more complex mechanisms between the above variables. This study aims to explore the co-existing impacts of parenting style and self-esteem on rejection sensitivity.

**Methods:**

It is a cross-sectional study and 1341 Chinese college students completed a questionnaire on parenting style, rejection sensitivity, and self-esteem. Latent profile Analysis (LPA) would be used to examine the model.

**Results:**

The profiles of parenting effecting self-esteem showed a three-solution profile. Profile 1 (high parental control group) received highest parental control and lowest self-esteem. Profile 2 (medium parental control group) had moderate level of parenting styles and self-esteem. Profile 3 (low parental control group) had the lowest parental control, highest care, encouragement, and self-esteem. The profiles of parenting and self-esteem effecting rejection sensitivity also showed a three-solution profile. Profile 1a (high parental control and low self-esteem group) showed highest rejection sensitivity. Students in Profile 2a (low parent control and high self-esteem group) showed lowest rejection sensitivity. Students in 3a (moderate parental control and self-esteem group) received moderate levels of rejection sensitivity.

**Conclusion:**

Current research has enriched our understanding of whether parenting styles and self-esteem coexist and how they affect rejection sensitivity. Provide empirical support to guide parents in educating their children, helping children improve self-esteem, and reducing rejection sensitivity.

**Supplementary Information:**

The online version contains supplementary material available at https://doi.org/10.1186/s40359-026-05240-8.

## Introduction

Rejection sensitivity refers to the tendency of an individual to have anxious expectation that he will be rejected by others in the process of social interaction, as well as the preparatory perception and overreaction behavior to rejection cues in emotion or behavior [49, 67]. Research on rejection sensitivity is particularly important among Chinese university students. Firstly, influenced by Confucian values and collectivism, Chinese families tend to place greater emphasis on parental authority and adopt more controlling parenting practices compared to their Western counterparts [44, 59]. In such an environment, children may become more sensitive to rejection and more eager for social approval, which arguably amplifies the negative impact of rejection sensitivity on individuals [44]. Additionally, university students are undergoing the developmental transition from adolescence to adulthood [42]. During this period, they gradually become more independent from their families and begin to establish increasingly complex interpersonal networks, and become more sensitive to internal rejection and acceptance [42]. Previous research has shown that rejection sensitivity is closely associated with psychological well-being and social adjustment [10]. Individuals with high levels of rejection sensitivity are more likely to experience psychological difficulties such as depression, loneliness, and anxiety, and to exhibit more maladaptive cognitive and behavioral patterns in social interactions [41, 50].

Specifically, highly rejection-sensitive individuals tend to anxiously anticipate rejection from others and are more likely to interpret ambiguous social cues as signs of rejection [10, 20, 50]. As a result, they may experience greater difficulties in emotion regulation and are more likely to engage in avoidant or hostile behaviors during interpersonal interactions [10, 20, 50]. These maladaptive responses may in turn elicit more negative social feedback, contributing to lower well-being and poorer interpersonal relationships [10]. Therefore, in order to help college students develop better, it is very necessary to study the rejection sensitivity of college students.

A large number of theoretical reasoning and empirical studies have shown that parenting style is an important factor predicting individuals’ rejection sensitivity. Parenting style refers to a relatively stable pattern in the daily life of attitudes and behaviors that parents adopt when raising and educating children. It reflects parents’ parenting concepts, parenting behaviors and emotional expressions, and reflects parents’ education concepts and emotional attitudes regarding their children [54].

The theory of the influence of parenting style on rejection sensitivity has been confirmed by many studies in recent years. The study on adolescents found that when parents adopt negative parenting styles such as rejection and control, children feel neglected or controlled, which increases their rejection sensitivity level [26, 68]. On the contrary, emotional warmth and encouraging autonomy make children feel respected and accepted, and individuals expect and perceive less rejection experience, which is significantly negatively correlated with rejection sensitivity [63]. Studies on college students also prove that moderate parental care can reduce children's rejection sensitivity [21]. Additionally, a recent cross-cultural longitudinal study demonstrates that adolescents' subjective perceptions of parental warmth are more strongly associated with internalizing symptoms than parental self-reports, suggesting that adolescent-reported parenting has unique predictive validity beyond objective parental behavior [15]. This provides methodological support for the current study's use of adolescent self-reported parenting styles.

However, if it exceeds a certain degree, parents' excessive preference and control will also make children lack a certain degree of independence, resulting in a lack of security in social interaction, and fear of being negated and punished by others [60]. In addition, some research has further confirmed that parent-adolescent pattern with high control and low autonomy support is closely related to poorer adolescent psychological health and higher levels of adolescents’ internalizing problems, including anxiety and depressive symptoms [39, 60]. In a study with a sample of 500 students, authoritative and authoritarian parents, characterized by “high control”, have increased restrictions on their children's autonomous behavior compared to the control group, and children tend to feel rejected, depressed and hurt easily, with significantly higher rejection sensitivity [28]. While steeper declines in perceived maternal overparenting predicted lower anxiety and depressive symptoms over time [28]. But the neglectful parenting style characterized by “low control” can also increase children's rejection sensitivity [14]. These studies offer support for the hypothesis that profiles characterized by high control and low autonomy encouragement will be associated with poorer outcomes. By contrast, high care and encouraged parenting styles are more conducive to reducing children's rejection sensitivity.

According to a rejection sensitivity model based on attachment theory [4, 5, 11], rejection sensitivity is the result of an individual's early rejection experience and affects the individual's psychosocial function [16]. When parents ignore or refuse children's needs, children will develop insecure attachment patterns in the long run, including doubt and worry about whether others accept and support them [32]. This rejection experience has formed a long-term cognition, that is, they are prone to be rejected by others when expressing their needs, and after refusing, they cannot meet their needs of care and support. Therefore, they learn to pay special attention to avoiding rejection by others and feel anxious about expectations when expressing their needs. As a result, they are overly sensitive to rejection cues, and are more likely to experience rejection, which in turn produces emotional or behavioral overreactions [12]. At the same time, the parental acceptance-rejection theory also proves that children who are severely rejected by their parents (neglect, indifference, hostility, etc.) in the early stage will feel extreme pain and resentment, and from then on, the former individuals cannot easily trust others in the new relationship, which is manifested as a high level of rejection sensitivity [9, 25].

One reason for this may be self-esteem. Previous studies have also found that the influence of parenting style on rejection sensitivity may be affected by self-esteem. Excessive protection and control of children will make children deny themselves to a certain extent, thus losing self-confidence, fearing being evaluated and abandoned by others, resulting in high rejection sensitivity [7]. Self-esteem is an individual's attitude and evaluation of self-ability and value, and is an important factor influencing individual social behavior [51]. On the one hand, parents, as important others of their children, have an important influence on the formation and development of individual self-esteem [18]. Studies have shown that the warmth and care of parents are more conducive to the formation of high self-esteem of children, while parenting styles such as rejection and excessive interference are closely related to children's low self-esteem [1, 23]. At the same time, parental autonomy support and psychological control have a significant impact on self-esteem, support autonomy positively predicts self-esteem, and parental control has an inhibitory effect on self-esteem [56]. On the other hand, individuals with low self-esteem tend to show higher rejection sensitivity [13, 43, 64]. Due to long-term rejection by parents and society, an individual's sense of self-value is destroyed [27], thus forming a negative self-concept, which is manifested as low self-esteem, lack of self-confidence, and longing for affirmation and support from others [34]. Therefore, special attention is paid to avoiding rejection, but at the same time, more attention is paid to negative feedback. They tend to produce strong attention bias for rejection cues [3], which is reflected in high rejection sensitivity [55, 69].

In summary, both parenting style and self-esteem have an impact on rejection sensitivity, and there may be more underlying mechanisms between these variables. Therefore, latent profile analysis (LPA) would be a suitable method. LPA can generate profiles with similar levels of parenting style dimensions and self-esteem, aiming at exploring whether the coexistence of parenting style and self-esteem will affect the rejection sensitivity of college students as a special group. Based on the above theoretical viewpoints and empirical research, hypotheses are proposed: (1) The dimensions of control, care and encouragement in parenting style will influence students' self-esteem development; (2) Profiles of the three dimensions of parenting style will coexist with different levels of self-esteem (i.e., high, medium, and low); (3) Profiles with a high degree of parental control will exhibit higher rejection sensitivity than other profiles.

## Methods

### Participants and procedures

Using the convenient sampling method, this study recruited the full-time undergraduate students from universities in Dongguan, Guangdong Province, China, between March and April 2024. Participants were recruited through completed electronic questionnaires in class. To reduce potential sources of bias, this study is voluntary and anonymous. Participants have been informed of the research purpose and requirements before filling in the questionnaire, and have the right to withdraw from the study at any time without providing reasons. Questionnaire were collected immediately after filling in the class, and the research data kept fully confidential.

The inclusion criteria for participant recruitment were as follows: 1) full-time college student, and 2) age 18–23. The exclusion criteria were: 1) incomplete questionnaire data, 2) obviously invalid responses, and 3) failure to meet the age or student-status requirements.

A total of 1593 questionnaires were sent out and collected, and 1341 valid questionnaires were retained after weeding out invalid questionnaires such as incomplete information, with an effective recovery rate of 84.2%. A total of 1341 participants were enrolled in the study, with an average age of 18.4 ± 0.61 years old.

### Measures

Parenting style was assessed using the Chinese revision of the Parental Bonding Instrument (PBI) developed by Yang et al. [65] based on the original instrument by Parker et al. [46]. The revised version includes separate forms for fathers and mothers, with 23 items in each form. Both forms assess three dimensions of perceived parenting: care, control, and encouragement of autonomy. Items were rated on a 4-point Likert scale (ranging from 0 = very unlike to 4 = very like), with higher scores indicating higher levels of the corresponding parenting characteristic. Higher care scores reflect greater parental warmth, understanding, and emotional support,higher control scores reflect more intrusive regulation and restriction,and higher encouragement-of-autonomy scores reflect greater support for independent problem solving and self-direction.

Previous research has shown that the Chinese revised PBI has acceptable reliability and validity in Chinese samples. In the present study, internal consistency was good for both the maternal and paternal forms. For the maternal form, Cronbach’s alpha coefficients were 0.880 for care, 0.860 for control, and 0.730 for encouragement of autonomy. For the paternal form, the corresponding coefficients were 0.932, 0.910, and 0.857. In addition, correlations among the PBI subscales were in the expected directions, and their associations with self-esteem and rejection sensitivity were also theoretically consistent, providing preliminary support for construct validity in the present sample.

Rejection sensitivity was measured with the Chinese version of the Rejection Sensitivity Questionnaire (RSQ), originally developed by Downey and Feldman [11] and later revised for use in Chinese samples by Sun Xiaoling and colleagues. The questionnaire adopted a six-point score, and 16 situations were set up, which were evaluated from two dimensions: 1) anxiety level of rejection. The higher the score, the more anxious and worried the subjects were about rejection,And 2) the level of anticipation of acceptance. The higher the score, the more likely the subject expected others to accept their request. Rejection sensitivity score = Rejection anxiety inverse score of acceptance expectation, with higher scores indicating higher rejection sensitivity. The internal consistency reliability of the scale was 0.835 [11]. In this study, its internal consistency reliability was 0.890.

Self-esteem was assessed using the State Self-Esteem Scale (SSES) developed by Heatherton and Polivy [24]. The scale consists of 20 items with three related factors: performance, social, and appearance self-esteem. A seven-point scale was used from 1 (not at all) to 7 (extreme), with higher overall scores representing higher levels of self-esteem. The scale has good reliability and validity, and its internal consistency reliability is 0.920. In this study, its internal consistency reliability is 0.930.

### Statistical analysis

Descriptive statistics were used in this study to describe the sample. Correlation coefficients were used to establish the relationship between population variable and the outcome variable. Mplus7.4 was used to conduct latent profile analysis (LPA) to determine the profile of rejection sensitivity of college students under the combined influence of different parenting styles and self-esteem. The LPA analysis covered three dimensions of parenting styles of father and mother respectively (paternal care, paternal control, paternal encouraging autonomy, maternal care, maternal control, and maternal encouraging autonomy), rejection sensitivity, and self-esteem. Models with k = 1 through k = 5 profiles were estimated sequentially. The best profile solution was determined by evaluating Akaike Information Criterion (AIC), Bayesian Information Criterion (BIC), sample size adjusted BIC (ssaBIC), Bootstrap likelihood ratio test (BLRT), Lo-Mendell-Rubin (LMR), and entropy value. Lower AIC, BIC and ssaBIC values indicate better overall model fit. The BLRT and LMR were used to compare adjacent models, and significant *p* values (*p* < 0.05) indicated that a k-profile model was significantly better than the k-1 profile model. Entropy would be used to assess the accuracy of the classification. An entropy greater than 0.8 indicated a classification accuracy greater than 90% and was considered acceptable [8, 35, 45]. When the fit indices pointed to different solutions, theoretical interpretability and model parsimony were additionally considered in the final selection. In addition, profile group would be excluded when it was less than 5% of the sample.

## Results

### Descriptives and correlation

The participants (*N* = 1341) were full-time college students from Guangdong, China, of whom 390 were male (29.1%) and 951 were female (70.9%). Among the families, 92.2% were two-parent families, 7.5% were single-parent families, and 0.4% had no parents to raise them. Regarding intimate relationships, 73.6% had no experience, 13.3% were currently in an intimate relationship, and 13% had been in an intimate relationship but returned to being single.

According to the scoring methods of the measurement of PBI, this study explored the distribution of factors of family parenting style: 29.5% of the participants received high maternal care, 17.2% received low maternal care; 8.0% had high maternal control level and 30.6% had low maternal control level; 33.0% received high paternal care, 14.3% had low paternal care, 8.1% had high paternal control and 36.6% had low paternal control. Further analysis found that accross all parenting dimensions, the proportion of father and mother with the same parenting style was as high as 97.1% of participants, in which the parents providing high care and moderate control accounted for the most, 98.8% and 61.8% respectively. This result suggests that in Chinese college students' families, both parents have the same parenting style for their children, and more parents tend to give their children a high level of care and moderate control.

Table 1 showed the correlation coefficients among the various variables in this study. Specifically, paternal and maternal care and encouraging autonomy were significantly positively correlated with self-esteem, and negatively correlated with rejection sensitivity (all *p* < 0.01); Paternal and maternal control were significantly negatively correlated with self-esteem and positively correlated with rejection sensitivity (all *p* < 0.01); There was significant negative correlation between self-esteem and rejection sensitivity (all *p* < 0.01).

**Table 1:** Correlations between variables

	1	2	3	4	5	6	7	8	9	10	11	12
1.Gender	1.000											
2.Age	−0.080**	1.000										
3.IR	−0.044	0.005	1.000									
4.FS	0.057*	0.048	0.054*	1.000								
5.FCare	−0.065*	−0.035	0.010	−0.154**	1.000							
6.FEnco	−0.025	−0.008	0.018	−0.083**	0.614**	1.000						
7.FCon	0.033	0.026	−0.031	−0.007	−0.339**	−0.429**	1.000					
8.MCare	−0.057*	−0.028	0.005	−0.092**	0.993**	0.520**	−0.361**	1.000				
9.MEnco	−0.015	0.010	0.024	−0.022	0.504**	0.824**	−0.400**	0.615**	1.000			
10.MCon	0.027	−0.006	−0.016	0.018	−0.373**	−0.425**	0.822**	−0.392**	−0.485**	1.000		
11.SelfE	−0.025	−0.005	0.072**	−0.067*	0.351**	0.312**	−0.191**	0.326**	0.304**	−0.202**	1.000	
12.RS	−0.012	−0.004	−0.025	0.040	−0.280**	−0.288**	0.176**	−0.269**	−0.276**	0.170**	−0.517**	1

*IR* Intimate relationships, *FS* Family status, *FCare* Father care, *FEnco* Father encourage, *FCon* Father control, *MCare* Mother care, *MEnco* Mother encourage, *MCon* Mother control, *SelfE* Self-esteem, *RS* Rejection sensitivity

^**^p <.01

^*^p <.05

### Latent profile analysis

The goodness of fit indexes of the continuous potential class models are shown in Tables 2 and 3 respectively, and two to five profile solutions are set for latent profile models fitting estimation in both groups of profile models.

**Table 2:** Comparing psychological constructs between parenting styles and self-esteem groups

Profile	Log-likelihood	AIC	BIC	SSA-BIC	Entropy	BLRT p	LMR-LRT p	Smallest class size
Profile 2	−10,088.699	20,215.399	20,314.221	20,253.866	0.860	<.001	<.001	37%
Profile 3	−9676.156	19,404.313	19,539.543	19,456.953	0.877	0.003	0.004	11%
Profile 4	−9480.603	19,027.207	19,198.845	19,094.019	0.865	0.200	0.207	10%
Profile 5	−9264.781	18,609.562	18,817.609	18,690.546	0.879	0.119	0.122	2%

*AIC* Akaike Information Criterion, *BIC* Bayesian Information Criterion, *SSA-BIC* Sample-Size Adjusted BIC, *BLRT* Bootstrap Likelihood Ratio Test

**Table 3:** Comparing psychological constructs between parenting styles, self-esteem and rejection sensitivity groups

Profile	Log-likelihood	AIC	BIC	SSA-BIC	Entropy	BLRT p	LMR-LRT p	Smallest class size
Profile 2	−11,922.673	23,889.347	24,003.773	23,933.888	0.86	<.001	<.001	38%
Profile 3	−11,493.292	23,046.583	23,202.619	23,107.322	0.876	0.003	0.003	11%
Profile 4	−11,294.578	22,665.156	22,862.8	22,742.091	0.867	0.142	0.147	10%
Profile 5	−11,073.397	22,238.794	22,478.048	22,331.926	0.879	0.037	0.039	1%

*AIC* Akaike Information Criterion, *BIC* Bayesian Information Criterion, *SSA-BIC* Sample-Size Adjusted BIC, *BLRT* Bootstrap Likelihood Ratio Test

**Fig. 1:**
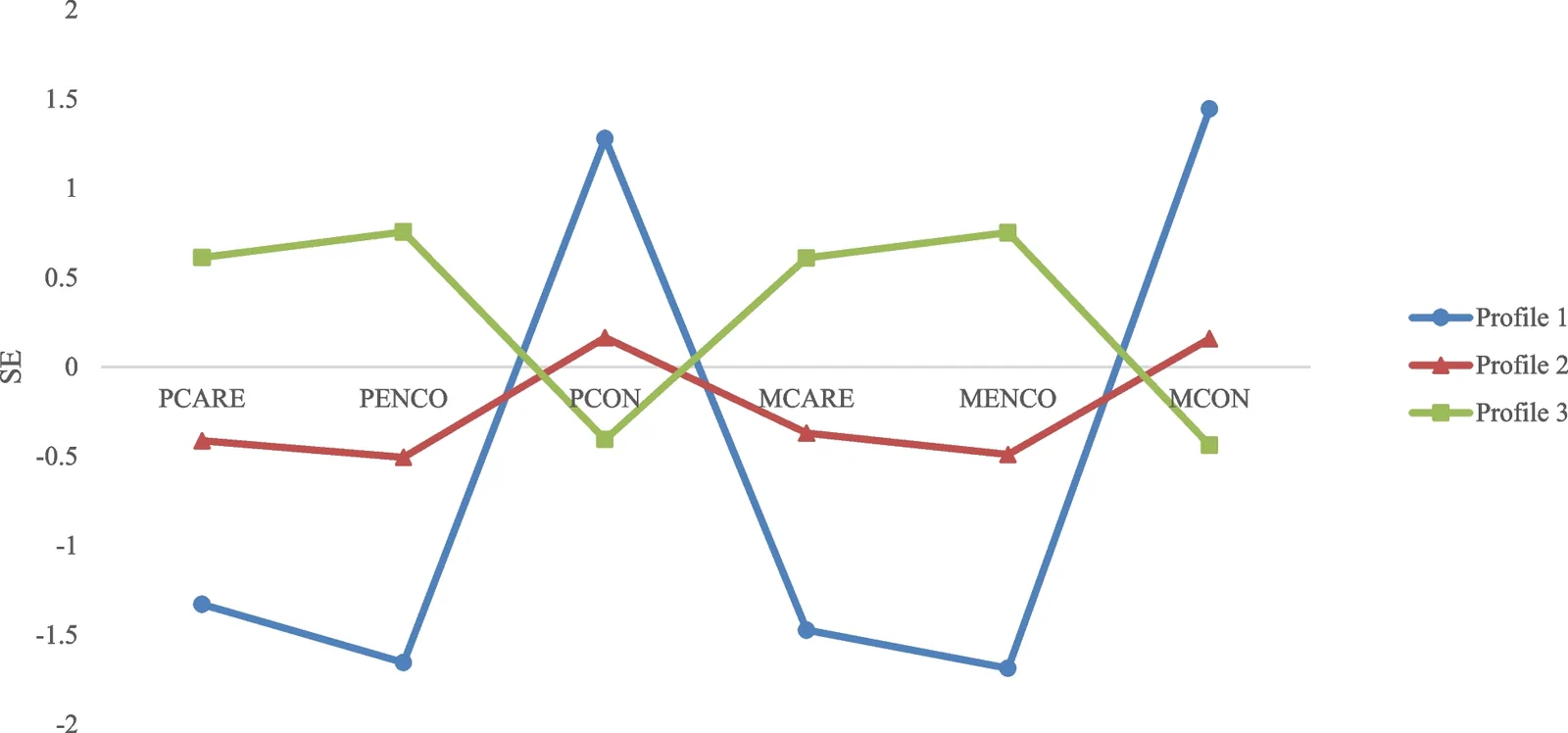
Latent profiles of participants with parenting styles. Note: FCARE = paternal care; FENCO = paternal encouraging autonomy; FCON = paternal control; MCARE = maternal care; MENCO = maternal encouraging autonomy; MCON = maternal control; SelfE = self-esteem

First, the study explored the differences in student self-esteem development among various parenting style groups. Table 2 showed the increasing numbers of profiles, the values of AIC, BIC and ssaBIC gradually decreased, and the amplitude of decrease gradually slowed down after examining the three-profile solutions with high entropy values, indicating that three profile solutions is superior to two and four profile solutions. At the same time, the *p*-values of BLRT and LMR in three profile solutions were significant (both *p* < 0.01). In addition, from the results of the proportion of the smallest group, it can be seen that the smallest group size of five profile solutions was less than 5% of the total number of participants, which indicated that three profile solutions were superior to five profile solutions. Therefore, three profile solutions were selected as the optimal model. More specifically, in Profile 1 (high parental control group), students received high levels of paternal and maternal control, but low levels of paternal and maternal care and encouraging autonomy. The students in Profile 2 (moderate parental control group) had moderate levels of paternal and maternal control, care, and encouraging autonomy. Students in Profile 3 (low parental control group) had the lowest level of parental and maternal control, but had higher levels of paternal and maternal care and encouraging autonomy (See Fig. 1).

The difference test of the three groups of three profile solutions showed that there were significant differences among each group (all *p* < 0.01). Among them, Profile 3 had the highest value (Mean = 0.343, S.E. = 0.041), and its self-esteem level was the highest among the three profiles, while the self-esteem level of Profile 1 was significantly lower than that of the other two profiles (Mean = −0.627, S.E. = 0.083). The self-esteem of Profile 2 was in the middle (Mean = −0.263, S.E. = 0.040).

**Fig. 2:**
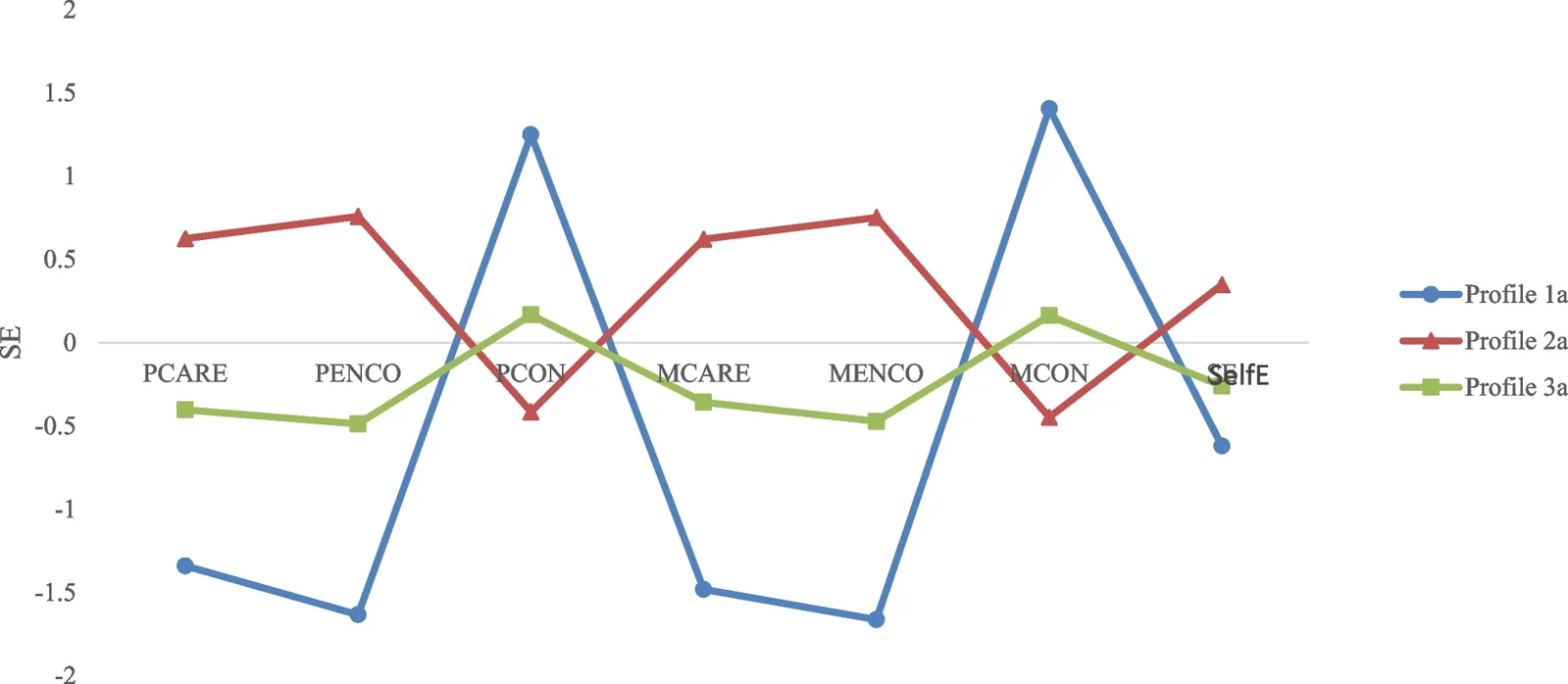
Latent profiles of participants with parenting styles and self-esteem. Note: FCARE = paternal care; FENCO = paternal encouraging autonomy; FCON = paternal control; MCARE = maternal care; MENCO = maternal encouraging autonomy; MCON = maternal control; SelfE = self-esteem

After determining the first profile model, the study also explored differences in rejection sensitivity among groups with different parenting styles and self-esteem development, with the results shown in Table 3. According to the decreasing trend of AIC, BIC and ssaBIC values, it can be found that the three profile solutions have the largest decreasing amplitude, indicating that the three profile solutions are more suitable than the two and four profile solutions. Moreover, the p values of BLRT and LMR of the three profiles were significant (both *p* < 0.01). In addition, the smallest group size of the fifth profile was only 1%, which again indicated that the three profile solutions were better than the five profile solutions. As a result, the final model was set to a three-profile solution, which was considered optimal. That is, in Profile 1a (the high parental control and low self-esteem group), students were subject to high levels of paternal and maternal control, but low levels of paternal and maternal care and encouraging autonomy, and demonstrated low levels of self-esteem. In contrast, students in Profile 2a (the low parental control and high self-esteem group) had the lowest levels of paternal and maternal control, the highest levels of paternal and maternal care, encouraging autonomy, and high self-esteem. Students in Profile 3a (the moderate parental control and self-esteem group) received moderate levels of parental control, care, and encouraging autonomy, and had moderate levels of self-esteem (See Fig. 2).

According to the results in difference test of the three groups, there were significant differences among each group (all *p* < 0.01). The rejection sensitivity of Profile 1a was significantly higher than that of the other two groups (Mean = 0.627, S.E. = 0.095), and the rejection sensitivity of Profile 2a was the lowest (Mean = −0.316, S.E. = 0.037). In Profile 3a, rejection sensitivity was moderate (Mean = 0.218, S.E. = 0.046).

## Discussion

This study examined how parenting styles and self-esteem co-occur in relation to rejection sensitivity among Chinese college students using a latent profile approach. First, profiles were generated using three parenting styles of mother and father as observed variables to examine the levels of self-esteem. Second, three parenting styles of mother and father and self-esteem as observed variables were generated profiles to examine the levels of rejection sensitivity. The characteristics and performance of these profiles of students support the three hypotheses of this study.

The finding about the parenting styles profiles is mainly characterized by parental control level, with the father and mother showing highly similar patterns across dimensions. It is worth noting that in the parenting of Chinese college students in the present study, the style adopted by parents is 97.1% consistent in dimension and level. That is, when one of parent gives the child high control and low care, another one will also adopt the same parenting style. This result supports a family-systems perspective, suggesting that parenting behaviors within the same family may be coordinated rather than independent. This may be because people tend to mate with people who behave in a similar way to themselves [36]. Then, the behavior patterns of one parent are also influenced by the other parent and by both [37, 66]. These results support the need to consider the family as a system and explore the co-existence of paternal and maternal parenting styles. The high level of similarity between maternal and paternal parenting may reflect shared family norms, mutual influence between parents, or the possibility that one parent’s approach shapes the other’s behavior over time.

Turning to the effects of profile solution on self-esteem, students in the high control group (profile 1) had significantly lower levels of self-esteem than the other two groups. In contrast, students who received lower parental control and higher levels of care and encouraging autonomy (profile 2) had higher self-esteem, while those who received lower parental control and higher care and encouraging autonomy achieved the highest levels of self-esteem (profile 3). In essence, as an important place for children's socialization, family is the first and most important external factor affecting children's self-esteem development [48]. As the important others of children, the attitude and standards of parents affect the child's judgment of self [52]. This pattern is consistent with theories of psychological control and autonomy support, which propose that high control, particularly when combined with low warmth or low autonomy encouragement, may undermine self-worth, whereas warmth and autonomy support may foster more positive self-evaluations. Recent studies have found that parents' care and encouragement are the key to children's positive self-evaluation. Children with high parental care tend to have higher autonomy, and are more likely to form independent and confident personalities in continuous self-exploration [47]; Parents who adopt high control often fail to give enough love and encouragement, leaving their children in denial and rejection. This pattern has been associated with increasingly negative self-concept, including feelings of being failures, of having made mistakes, and unworthy of love, and thus showing low self-esteem [6, 57]. Recent longitudinal evidence also suggests that perceived overparenting is not fixed in its effects: declines in perceived overparenting have been linked to improvements in adolescent well-being over time [28]. This finding is relevant because it implies that the negative associations observed in the present study may be modifiable rather than immutable.

Regarding the co-existing effects of parenting styles and self-esteem on rejection sensitivity, students in the profiles characterized by higher parental rejection and lower self-esteem groups (profile 1a) reported the highest levels of rejection sensitivity. This pattern is consistent with attachment-based accounts of rejection sensitivity and with the parental acceptance-rejection framework, which suggest that repeated experiences of low acceptance, criticism, or control may be associated with heightened expectations of rejection in later interpersonal contexts. On the one hand, excessive parental control may deprive children of opportunities to explore new environments and develop independent personalities by increasing their perception of rejection threats [30, 38]. This may shape the child's general expectations of whether they will be accepted, rejected, supported, or ignored by others [19, 53].

In this process, children may form negative self-evaluation in the constant denial and rejection of parents, and habitually pay more attention to rejection cues [3, 62]. At the same time, children with low self-esteem show a stronger neurophysiological response to rejection, and this adverse cognitive and physiological response also strengthens the expectation of rejection threat [17]. On the other hand, parents lack care and encouragement for their children, and even show indifference and hostility, which may be associated with children’s anxiety of not being loved and constantly alert to cues of parents' rejection [2, 69]. This kind of negative parenting is seen as an attack on the child's self-esteem. Children with low self-esteem desire to be accepted, can only try to conform to the parents, and also strengthen the child's rejection sensitivity and associated anxiety [22]. Furthermore, Leung and Shek [28] found that declines in perceived parental control over time were associated with improvements in individuals' psychological wellbeing. This finding supports the notion that the negative effects of high parental control may be amenable to change [28]. It also points to meaningful directions for practical intervention, suggesting that reducing parental control could serve as an effective approach to enhancing self-esteem and alleviating rejection sensitivity.

In contrast, as the group with the lowest rejection sensitivity, profile 2a students received both the lowest parental control and the highest care and encouraging autonomy, and exhibited the highest levels of self-esteem. This result is supported by a cross-sectional study in which students who received positive parenting styles showed lower rejection sensitivity [40]. An open and autonomous growth environment allows children to face and deal with more rejection situations. At the same time, parental warmth and understanding can help children achieve stable social adjustment and emotional functioning, and also effectively reduce anxiety in the face of possible rejection [70]. This interpretation is further supported by observational research by Wentholt et al [60], which used observational rather than self-report method. The convergence of findings across observational method and self-report method strengthens confidence in the role of autonomy support as a factor associated with lower rejection sensitivity. Furthermore, children with higher self-esteem had more positive and stable self-evaluations, which played a protective role in reducing rejection sensitivity [29].

Notably, there was no evidence in the two high-care profiles (Profile 3 & Profile 2a) that excessive care actually lowered self-esteem and increased rejection sensitivity. This is not consistent with previous findings. According to Li et al. [30], excessive preference and protection would make children overly dependent on their parents, lose self-confidence, and be sensitive to others' speech and behavior in social situations. In this study, however, students showed high self-esteem and low rejection sensitivity despite receiving high care. This suggests that the reason may be that the care students received had not reached a high enough level to have the opposite effect on self-esteem and rejection sensitivity. The impact of excessive care can be further examined in the future.

In addition to high and low parental control, profiles characterized by medium control (profile 2 & Profile 3a) deserve special attention. Their results reflect the influence of certain characteristics of parenting styles on rejection sensitivity and self-esteem. Family experiences may not fall neatly into purely adaptive or maladaptive categories. Rather than overinterpreting this profile as reflecting a single family process, it is more prudent to view it as a heterogeneous pattern that may emerge from different combinations of parental warmth, control, and autonomy support. Compared with the other two parenting styles, these students did not seem to feel too much attention and control from their parents, and their performance was not outstanding in every dimension. This is in line with the mixed parenting style proposed by Peng et al. [61]. Specifically, parents of such students may be forced by life pressure and other reasons to focus more on their careers, leading to neglect of their children's needs. When they become aware of neglect, they may compensate for the lack of attention by meeting greater needs [31]. But in turn, they may also save time on education through harsh punishments and restrictions [33]. Parent–child processes can be bidirectional. For example, Bai et al. [39] reported that adolescent characteristics may also shape parenting behaviors over time, implying that children are not merely passive recipients of parenting. In this sense, the moderate profile may reflect a dynamic family relationship that cannot be fully captured by the present study’s one-time assessment.

The present findings also have practical implications. Because the high-control profile was associated with lower self-esteem and higher rejection sensitivity, interventions should not only focus on reducing harsh control but also on increasing autonomy support and emotional warmth. At the same time, the finding that lower perceived overparenting may relate to better outcomes suggests that parenting interventions may be beneficial even in later adolescence and emerging adulthood. However, such implications should be understood as tentative and hypothesis-generating rather than definitive, given the observational design of the study.

Overall, this study contributes to the literature by showing that parenting styles and self-esteem form distinguishable profiles that are meaningfully associated with rejection sensitivity. The findings are broadly consistent with attachment-based and self-evaluative theories, but they do not establish causal direction. Instead, they suggest that profiles marked by high control and low self-esteem may represent a psychosocial risk configuration, whereas profiles marked by higher care, greater autonomy support, and higher self-esteem may represent a relatively protective configuration.

### Limitation

It should be admitted that there are some limitations in this study. First of all, in terms of the sample itself, the participants are all Chinese college students. But the issue of rejection sensitivity does not only exist in this group, so there are certain limitations in terms of region and age. It remains to be seen whether the results of this study can be generalized to college students in other cultural contexts, including Western countries. Second, the study did not exclude participants from non-two-parent families. Although such participants were a minority of the overall population (7.9%), when completing the PBI, such participants were informed that they could substitute their closest same-sex dependent as a parent. But this sample bias may affect the generality of the findings. Moreover, because the variables in this study involve multiple types and dimensions, participants need to fill in more questionnaire items. Therefore, the results of the study may have been affected by the fatigue problem. In future studies, attention should be paid to avoid making participants face a large number of questions. At the same time, the importance of questionnaires and the establishment of certain reward mechanisms should be emphasized to improve the concentration of participants. Another limitation concerns the potential for common method bias. All major variables in the present study, including perceived parenting style, self-esteem, and rejection sensitivity, were assessed using self-report questionnaires collected at the same time point. Therefore, the observed associations may have been influenced by shared method variance, participants’ response styles, recall bias, or social desirability.

Finally, this study cannot establish the causal associations among parenting styles, self-esteem, and rejection sensitivity beacuse of its cross-sectional design. A recent longitudinal study using a Chinese adolescent sample found a bidirectional effect between parental autonomy support and internalizing problems [39]. This study indicated that rejection sensitivity may also influence parenting style in turn. Additionally, the research on the stability of adolescents’ profiles over three years finds that individuals’ profile membership can change over time [58]. Therefore, the longitudinal stability of parenting style, self-esteem and rejection sensitivity cannot be guaranteed. In the future, longitudinal studies should be conducted to examine the developmental relationships and longitudinal stability of these three variables.

### Conclusion

This study shows that parenting styles and self-esteem co-occur in meaningful patterns that are associated with different levels of rejection sensitivity among Chinese college students. The findings extend existing research by suggesting that rejection sensitivity may be better understood as related to configurations of family relationship experiences and self-evaluative functioning, rather than to any single parenting dimension considered in isolation. In addition, the use of latent profile analysis provides a person-centered perspective that captures heterogeneity in how parenting and self-esteem cluster within individuals. These findings suggest that family environments characterized by lower control, greater care, and stronger autonomy support tend to co-occur with higher self-esteem and lower rejection sensitivity, highlighting potentially relevant targets for prevention and intervention. Overall, the study offers an empirical basis for future longitudinal research on how parenting experiences and self-esteem jointly shape vulnerability to rejection sensitivity across development.

## Supplementary Information

Supplementary Material 1.

## Data Availability

All data (e.g., datasets, survey instruments) generated or analysed during this study are included in this published article and its supplementary information files. Please see the link which is (link: https://pan.baidu.com/s/1RQRM6mZH7VE3EA7_yGNvcA?pwd=6ba5, code: 6ba5).
